# Evaluating the Human Risks of Consumption of Foods of Bovine Origin with Ivermectin Residues in Ecuador

**DOI:** 10.3390/foods13213470

**Published:** 2024-10-29

**Authors:** Valeria Paucar-Quishpe, Darío Cepeda-Bastidas, Richar Rodríguez-Hidalgo, Ximena Pérez-Otáñez, Cecilia Perez, Sandra Enríquez, Erika Guzman, Fernanda Ulcuango, Jorge Grijalva, Sophie O. Vanwambeke, Lenin Ron-Garrido, Claude Saegerman

**Affiliations:** 1 Research Unit of Epidemiology and Risk Analysis Applied to Veterinary Science (UREAR-ULiège), Faculty of Veterinary Medicine, University of Liege, 4000 Liège, Belgium; avpaucar@uce.edu.ec; 2 Fundamental and Applied Research for Animals & Health (FARAH) Center, Faculty of Veterinary Medicine, University of Liege, 4000 Liège, Belgium; 3 Instituto de Investigación en Zoonosis (CIZ), Universidad Central del Ecuador, Quito 170521, Ecuador; rrodriguez@uce.edu.ec (R.R.-H.); ximena.perezotanez@uclouvain.be (X.P.-O.); ienriquez@uce.edu.ec (S.E.); 4 Facultad de Ciencias Agrícolas, Universidad Central del Ecuador, Quito 170521, Ecuador; dacepedab@uce.edu.ec (D.C.-B.); ezguzman@uce.edu.ec (E.G.); fvulcuango@uce.edu.ec (F.U.); 5 Facultad de Medicina Veterinaria y Zootecnia, Universidad Central del Ecuador, Quito 170521, Ecuador; bcperez@uce.edu.ec (C.P.); jgrijalva@uce.edu.ec (J.G.); 6 Georges Lemaitre Centre for Earth and Climate Research, Earth & Life Institute, UCLouvain, 1348 Louvain-la-Neuve, Belgium; sophie.vanwambeke@uclouvain.be

**Keywords:** ivermectin residues, milk, meat, liver, urine, faeces, risk assessment, consumption

## Abstract

Ivermectin is a widely used antiparasitic in livestock, but its use can result in residues in bovine products and excretions. The objective of the present study was to determine the presence of ivermectin residues in cattle meat, liver, milk, faeces, and urine and assess consumer risk from chronic exposure through contaminated bovine products using a deterministic approach. To determine the presence of ivermectin residues, 124 samples were analysed by liquid chromatography. Residues were found in 68% of faeces samples and small percentages (3%) in liver, milk, and urine, with no residues detected in meat. The mean ivermectin residue in the liver (16.46 µg/kg) remained below the maximum residue limit (MRL); however, in milk (12.46 µg/kg), the residues exceeded the permitted MRL. The results obtained from chronic dietary exposure show that the consumption of ivermectin residues was low, and the risk was assessed as being rare to very rare. Additionally, this study reveals concerning levels of ivermectin residues in milk that may far exceed established safety limits. This situation emphasises the urgent need for stricter regulations and monitoring in milk production, particularly from small farms, to protect vulnerable populations. However, from a one health perspective, the presence of residues in faeces poses potential environmental hazards, warranting further research. Moreover, the detection of residues in milk, despite the ban on ivermectin use in dairy cattle, underscores the importance of compliance with food safety regulations and the need for continued vigilance in this area.

## 1. Introduction

The livestock sector plays a critical role in global food supply and security, providing essential nutrition through products like meat, milk, and eggs. These products account for 18% of global calorie intake and 34% of protein consumption, significantly enhancing human diets [1]. Meat and meat-derived products are an energy-dense source of high-quality protein enriched in micronutrients such as vitamin B12, iron, zinc, selenium, and phosphorus [2]. Milk and dairy products contribute significantly to calcium, phosphorus, iodine, riboflavin, and vitamins A and B12 [3]. In 2023, global milk production reached 965.5 million tons and meat production 76.6 million tons [4,5].

In Ecuador, the livestock sector is a vital component of the agricultural economy. The country has a cattle population of about 3.7 million heads [6], with 81% of this population raised by small producers who manage 20 or fewer cattle heads [7]. Cattle husbandry in Ecuador primarily relies on grazing, with about 80% located in tropical or subtropical areas. These areas create proper conditions conducive to diseases caused by endo- and ectoparasites, which are the leading causes of illness and production losses [8].

Various drugs are used to manage cattle internal and external parasites; one of the most widely used is avermectins. Avermectins are a class of macrocyclic lactones produced by the soil actinomycete *Streptomyces avermitilis* [9]. This drug was discovered in 1973 and introduced to massive commercial success in the animal health market in 1981 [10]. The most widely utilised derivative of avermectin is ivermectin. Five years after its introduction, it was sold in 46 countries and administered to 320 million cattle heads [11]. Its success in the livestock market is due to its strong activity against nematode and arthropod parasites. Ivermectin is used to treat billions of cattle heads, helping to boost the production of food and leather products, as well as keeping cattle healthy around the world [10].

**Figure 1:**
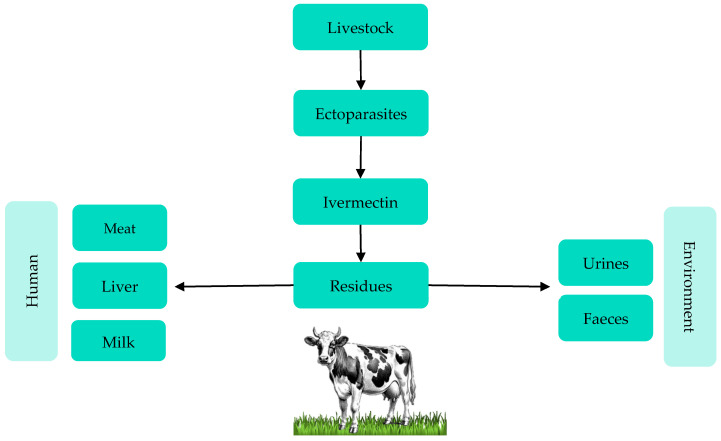
Interactions between ivermectin treatment, foods of bovine origin, the environment, and humans.

Although the health benefits of ivermectin are particularly important in ectoparasiticide control in livestock, its high level of faecal and urine excretion represents a potential environmental risk [12]. Additionally, the indiscriminate use of these drugs can result in trace amounts of residues and their metabolites persisting in edible tissues and animal products, such as meat, liver, and milk (Figure 1), which may pose potential health risks to people who consume these products [13].

To ensure that levels of acaricides and/or their metabolites in food of animal origin remain below thresholds which are hazardous to consumers, the livestock industry must strictly comply with mandated withdrawal periods to reduce residue levels. The Food and Agriculture Organisation of the United Nations (FAO) and the World Health Organisation (WHO) jointly administer Expert Committees on Food Additives (JECFA) that evaluate the safety of veterinary drug residues. These evaluations serve as the basis for national and international food safety standards set by the Codex Alimentarius Commission [14]. JECFA has introduced several measures for food safety assessment, including the maximum residue limit (MRL), which represents the maximum allowable level of veterinary drug residues in food of animal origin, the acceptable daily intake (ADI), indicating the maximum amount of a veterinary drug which can be consumed daily over a lifetime without appreciable health risks, and the global estimate of chronic dietary exposure (GECDE), which assumes that, in the longer term, an individual would be a high-level consumer of only one category of food and that their consumption of other foods containing the residue would remain at the population average [14]. The MRL for ivermectin allowed in foods of bovine origin to ensure that the ADI limit (10 µg/kg body weight per day) is not exceeded varies according to the type of tissue. The limits are 800 µg/kg in the liver, 400 µg/kg in fat, 100 µg/kg in the kidney, 30 µg/kg in muscles, and 10 µg/kg in milk [15]. However, in some countries, the MRL for ivermectin in milk is set to zero. Ivermectin is banned in milk production due to its highly lipophilic nature, which causes its residues to persist in milk and dairy products [16]. Therefore, in order to respect the withdrawal period, farmers must dispose of the milk and not slaughter the animal during the resting period, which represents an additional cost to tick control [17]. Dairy farmers continue to rely on the use of pesticides to control pests and increase productivity, looking only at the immediate advantages of pest control without considering the potential short- and long-term risks of residue accumulation.

This challenge is particularly acute for small dairy farmers, who must compete with larger producers that enjoy better market access and lower production costs. Compared to large industries, small farmers often grapple with daily issues such as inadequate infrastructure, limited resources, and insufficient access to veterinary care, making it difficult for them to produce high-quality milk at competitive costs [18]. In Ecuador, inconsistent supervision leads many small producers to operate in informal markets where quality controls are minimal [19]. Economic pressures and competition further exacerbate the situation, often compromising milk quality and posing potential health risks to consumers.

In this context, the objectives of this study were the following: (1) to determine the prevalence of ivermectin residues in small-scale dairy farms located in two subtropical areas of Ecuador in foods of bovine origin such as milk, meat, liver, and excretions like urine, and faeces; and (2) to assess the risk of consuming these foods of bovine origin through the measurement of ivermectin concentrations in these food items.

## 2. Materials and Methods

### 2.1. Study Area

This research was conducted in two livestock areas of Ecuador. Area 1, known as the “Northwest of Pichincha”, is situated in the province of Pichincha and includes the localities of Nanegal, Nanegalito, Pacto, Gualea, San Miguel de los Bancos, and Pedro Vicente Maldonado. This area is part of the Chocó Andino Biosphere Reserve, characterised by forests, rivers, waterfalls, and a diverse range of flora and fauna [20]. Area 2, known as the “Quijos River Valley”, is located in the province of Napo and comprises the localities of San Francisco de Borja, Sumaco, Linares, Sardinas, El Chaco, and Baeza. This area, situated in the foothills of the Andes Mountains and the high jungle of the Amazon region, is part of the protected areas of the Antisana Ecological Reserve, Cayambe Coca National Park, and Sumaco Napo Galeras National Park [21]. The residents in both areas are mainly involved in ecotourism, agriculture (tropical fruits, sugar cane, cacao, coffee, and palm hearts), fish farming (tilapia and trout), and cattle breeding [20,22,23].

Livestock in these areas primarily consists of small- and medium-sized cattle herds dedicated to dairy or dual-purpose production. The farmers in these areas frequently use dairy breeds such as Brown Swiss, Holstein, and Jersey or their crosses [22,24]. Feeding is mainly through grazing, but there is also the use of supplemental feeding based on concentrates or agro-industrial byproducts [18]. The cattle population in the study areas is around 100,000 heads, distributed across 4087 herds [25].

### 2.2. Sampling and Chemical Analysis

**Figure 2:**
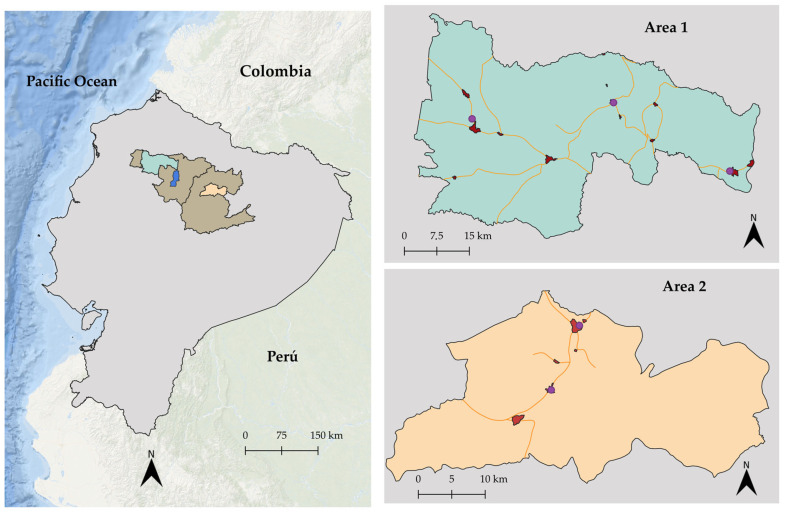
Location of study areas and sampling sites. Legend: 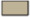 provinces of Pichincha (right) and Napo (left); 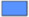 Quito (country capital); 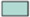 Area 1 (northwest of Pichincha); 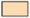 Area 2 (Quijos River Valley); 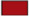 populated areas where surveys were conducted; 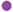 location of slaughterhouses; and 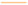 main routes of the milk collection trucks.

From 2021 to 2023, samples of milk, beef, and liver were collected from the two study areas. Raw cow milk samples (N = 70) were obtained from small milk tanks designated for collection. Each milk collector tank represented one farm and held approximately 40 litres from 5 to 7 cows. The selection of milk-tank samples was at the convenience of the researcher. It was based mainly on the farmer’s acceptance of participation in this study and the accessibility of the samples. Samples were collected following the primary route taken by the local milk collection trucks. Additionally, samples of urine (N = 39) and faeces (N = 40) were collected. Each of these samples consisted of a pool of 6 randomly selected cows from the farm. Meat (N = 46) and liver (N = 30) samples were collected at local slaughterhouses in each area. As one local slaughterhouse in Area 2 was closed, samples were acquired from a nearby slaughterhouse where animals from the study areas were relocated (Figure 2). The sampling process was carried out randomly, and official animal movement guides were reviewed to ensure that the animals were from the study areas.

Raw milk samples (100 mL) were collected in polyethene plastic vials, and the meat and liver samples (100 g) were stored individually in zip-lock plastic bags. All samples were kept in a cooler with ice blocks until they were transported to the laboratory. Analyses were conducted in the EcuaChemLab Chemical and Microbiological Laboratory of Ecuador, which is accredited according to NTE INEN ISO/IEC 17025 [26]. The concentration of the B1a component of ivermectin was analysed using a reverse-phase high-performance liquid chromatographic method. The analysis was conducted on a Perkin Elmer Series 200 HPLC system (Shelton, CT, USA) equipped with a JASCO UV-975 detector (Hachioji, Tokyo, Japan) and a ZORBAX Eclipse Plus C18 column. The limit of detection (LOD) was established at <10 µg/kg (Supplementary File S1).

### 2.3. Food Consumption Survey

A questionnaire was used to estimate milk, meat, and liver consumption. The questionnaire was validated by national and international experts in the field. It was pilot-tested with a small group of volunteers who commented on the clarity of the questions. The participants interviewed were male and female inhabitants of the populated parts of the study areas (Figure 2), heads of household, and over 18 years of age in 2024. The data were collected in paper-and-pencil format and contained questions on socio-demographic information (gender, age, and number of persons living in the household) and consumption habits of foods of bovine origin at the household level. The sample size was estimated using household data from the “Servicio Ecuatoriano de Normalización” (INEC) (2010) census [27], with a total of 17,194 households as the population size reference, a confidence level of 95%, and a margin of error of 5%. Consequently, the study included a sample size of 631 households. Information on beef, liver, and milk consumption was expressed in grammes, with the conversion factor of 1 mL of milk corresponding to 1.03 g.

### 2.4. Risk to Consumer Health

For human health, the Joint FAO/WHO Expert Committee on Food Additives (JECFA) has established for ivermectin an acceptable daily intake (ADI) for consumers of 0–10 µg/kg of their body weight [15].

Concentrations of ivermectin residues measured in milk, meat, and liver were compared to the maximum residue level (MRL) for human consumption. The MRLs are 10 µg/kg in milk, 30 µg/kg in muscle, and 800 µg/kg in liver [15]. There are several approaches for chronic exposure assessment and risk assessment. The World Health Organization (WHO) and the European Food Safety Authority (EFSA) recommend three scenarios for dealing with contamination data below the quantification limits. These approaches are named as follows: lower bound that induced underestimation (LB), middle bound that induced overestimation (MB), and upper bound that induced most overestimation (UB) [28,29]. In this study, we used the MB, whereby results below the limit of detection were replaced by LOD/2.

There is no international consensus on the age groups of consumers [30]. In this study, the risk was assessed for two groups—(1) individuals younger than 10 years and (2) individuals older than 10 years—in such a way that the latter group included adolescents and adults, following the recommendations of the American Academy of Pediatrics [31].

Two scenarios were analysed: (A) the overall study population and (B) specifically people who consumed foods of bovine origin.

The estimated daily intake (EDI) of ivermectin residues by the consumer was calculated as follows:

(1) $$EDI=\frac{contamination\;\left(\frac{mg}{g}\right)*consumption\;(g)\;}{bw\;(kg)}$$

where *contamination* is the mean concentration of ivermectin in the meat, liver, and milk; consumption stands for the daily mean *consumption* of these products in the study region; and bw represents the body weight. The mean *bw* in the study area for a person (man or woman) under 10 years of age was 13.50 kg (standard error (SE): 8.19 kg) and 68.39 kg (SE: 15.70 kg) for a person (man or woman) over 10 years of age. These data were obtained from a database provided by a local health clinic (N = 15,223).

The JECFA uses the global estimate of chronic dietary exposure (GECDE) for chronic dietary exposure assessment to veterinary drug residues [14]. According to the JECFA, ivermectin’s GECDE recommendation level for adults and the elderly is lower than 0.72 µg/kg bw per day, which represents 7.2% of the upper bound of the ADI of 10 µg/kg bw. The GECDE recommendation level for children is lower than 0.93 µg/kg bw per day, which represents 9.3% of the upper bound of the ADI of 10 µg/kg bw [15].

The Global Estimate Chronic Dose Exposure (GECDE) to ivermectin residues for the population in the study areas was the highest exposure calculated using the 97.5th percentile consumption figure for a single food selected from all the foods plus the mean dietary exposure from all the other relevant foods [32]:

(2) $$Highest\;exposure\;from\;each\;animal\;product={97.5\;}^{th}percentile\;consumption\;*\;Median\;residue$$

(3) $$Global\;Estimate\;Chronic\;Dose\;Exposure\;\left(GECDE\right)\;\;\;\;\;\;\;\;\;\;=Highest\;exposure\;from\;one\;animal\;product\;\;\;\;\;\;\;\;\;\;+Total\;mean\;exposure\;from\;all\;other\;products$$

## 3. Results and Discussion

### 3.1. Ivermectin Residues

**Figure 3:**
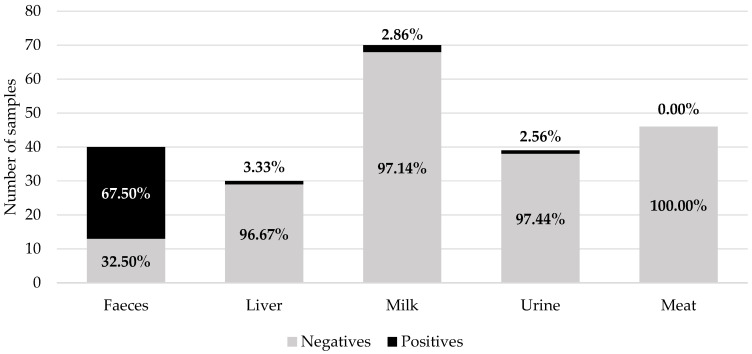
Prevalence of ivermectin residues in the analysed samples (decreasing order).

**Figure 4:**
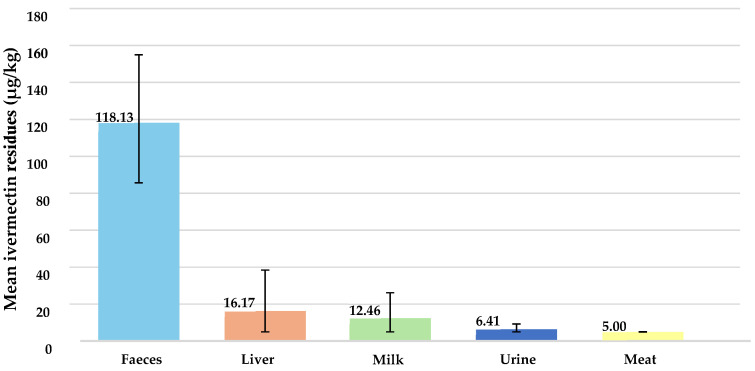
Mean of ivermectin residues in the analysed samples (decreasing order).

Of the total samples analysed (N = 225), the presence of ivermectin residues was determined in 68% of the faeces samples (27/40; values between 30 and 420 µg/kg) and around 3% in the liver (1/30; value of 340 µg/kg), milk (2/70; values of 90 and 440 µg/kg), and urine (1/39; value of 60 µg/kg) samples. No residue over the LOD was detected in the 46 meat samples (Figure 3 and Figure 4). Taking into account that the middle bound (MB) was used in this study, samples under the LOD (10 µg/kg) were assigned a value of 5 µg/kg. The mean value of ivermectin in faeces reached 118.13 µg/kg (95% CI: 85.61–155.00 µg/kg), 6.41 µg/kg (95% CI: 5.00–9.23 µg/kg) in urines, 12.46 µg/kg (95% CI: 5.00–26.16 µg/kg) in milk, 16.17 µg/kg (95% CI: 5.00–38.50 µg/kg) in liver, and 5.00 µg/kg (95% CI: 5.00–5.00 µg/kg) in meat.

This study was the first to detect ivermectin in foods of bovine origin such as milk, meat, and liver and excretions like urine and faeces from small-scale dairy farms. While HPLC did not detect residues above the maximum residue limit (MRL) in the meat and liver samples, the positive milk samples did exceed the MRL established by FAO [15]. Regionally, a study conducted in the Brazilian retail dairy market reported that ivermectin was substantially used in dairy cows; the authors reported that 46% of milk samples had some level of residues detected/quantified. Although these residues did not exceed the maximum residue limit (MRL), their presence in nearly half of the samples is concerning [33].

Although the Codex Alimentarius sets an MRL of 10 µg/kg for ivermectin, other regulatory bodies, such as the European Medicines Agency [34] and Health Canada (2024) [35], do not approve the use of ivermectin in dairy cattle, resulting in no established legal MRL for milk in these regions. Moreover, another study conducted in the areas of the present study indicates that ivermectin was used in dairy cattle at a rate of 50% [36]. The discovery that 68% of faeces samples contain ivermectin residues strongly confirms the widespread use of this drug among local farmers. Despite the fact that, in this study, the number of samples over 90 and 440 µg/kg, the MRL was small, and there are no additional studies conducted in the field where this has been studied, although there are several studies conducted on raw milk and meat, identifying the presence of antibiotics and heavy metals [37,38,39,40]. Additionally, some undergraduate research projects have conducted experimental studies investigating the occurrence and elimination of antiparasitics, such as eprinomectin, ivermectin, and fipronil, in meat and milk [41,42]. Freire [41], in 2017, evaluated the presence of fipronil and ivermectin residues in meat and found that the ivermectin levels (38 µg/kg) exceeded the permitted limit (30 µg/kg) [15] on day 15 post application (pour-on). Similarly, Balseca [42], in 2017, reported eprinomectin residues (19.06 µg/L) in milk close to the permitted limit (20 µg/L) [15] on day 19 after application (pour-on). Considering that two-thirds of the milk in Ecuador is marketed informally, where quality controls are minimal and represent the most accessible market for small producers [19], there is a pressing need for larger-scale studies to assess risk and ensure food safety and quality accurately. These comprehensive investigations are especially important given that milk is a fundamental food for children’s development, providing essential nutrients like calcium, protein, and vitamins [3]. A better understanding of the prevalence of ivermectin residues in milk is needed for informing regulatory decisions aimed at safeguarding public health.

While this study primarily focuses on the presence of ivermectin residues in faeces and urines, it is important to recognise the broader environmental implications. Low doses of ivermectin residues have been shown to cause significant short- and long-term ecological effects, including alterations in decomposer insect communities, the disruption of manure degradation processes, and changes in soil properties and functions [43,44]. Research has extensively examined the impact of ivermectin on decomposer insect communities, particularly dung beetles. It has been demonstrated that ivermectin can remain at toxic concentrations for insects for 28 days in pour-on treatments and 35 days in subcutaneous injections [45] and can persist in dung for as long as 180 days [46]. Studies indicate that adult beetles are attracted to dung with residues, leading to a 90% mortality rate when consuming fallen dung 2–3 days after injection [47]. Moreover, adult beetles that colonise dung with residues do not reduce the number of eggs laid, but the resulting larvae die early in their development [44]. The impact of residues on dung beetles is of particular interest due to their role in maintaining healthy pasture growth by facilitating dung removal, promoting aeration through tunnel formation, and facilitating nutrient recycling [43]. As a result, the effects of endectocide residues on soil degradation are an important consideration for the agricultural economy [44]. In addition, ivermectin residues may affect aquatic invertebrates, which are especially sensitive to its effects. In the case of ivermectin, the direct deposition of faeces from treated livestock into small watercourses, ponds, or lakes has been considered to be a major threat to aquatic ecosystems. Contamination through leaching has been considered unlikely, given that ivermectin is strongly adsorbed into soil and organic matter [48,49].

It is important to note that the absence of ivermectin residues in meat does not automatically ensure food safety. In our study, sampling was conducted in formal slaughterhouses with some level of sanitary control. However, in Ecuador, approximately 36% of foods of bovine origin come from informal slaughtering (clandestine or homemade) [50]. Furthermore, only larger farms have better access to official slaughterhouses, while smaller producers depend on intermediate dealers, who collect animals from different farms and transport them to livestock markets or directly to slaughterhouses [51]. Sampling local butcher shops could help determine the safety of the food reaching consumers.

### 3.2. Consumption of Foods of Bovine Origin

It was determined that most of the households surveyed consumed meat and milk (91% and 97%, respectively), but only 30% consumed liver. The daily consumption (DC) mean values of meat, liver, and milk, considering the two scenarios, are shown in Table 1.

Based on the survey data, the annual consumption in the study area was 10 kg of meat and 0.26 kg of liver for the whole population. There are no data available regarding the amount of liver consumption at the local or national level. However, meat consumption in our study was close to the national average of 13 kg per capita [52]. The national per capita consumption is similar to that of neighbouring countries like Colombia (14 kg) but lower than the reported consumption in South American countries such as Argentina (48 kg) or Brazil (35 kg), both of which are substantial consumers of meat globally [52].

Furthermore, our findings reveal an annual milk consumption per person of 32 litres, which is significantly lower than the national per capita average of 110 litres [53] and well below the recommended 180 litres by the FAO and OMS [54]. Previous studies in the country indicate that the highest per capita milk consumption is in the Highlands region of Ecuador. In contrast, in the Amazonian and coastal regions, where the study areas are situated, consumption is much lower, reaching a quarter of the consumption in the Highlands areas [55].

### 3.3. Risk Assessment for Consumers of Ivermectin-Contaminated Foods of Bovine Origin

The risk in this study was estimated based on the amount of ivermectin present in milk, liver, and meat, the consumption of these foods in the study areas, and the data evaluated by the JECFA [15].

For milk, 2 samples (90 µg/kg and 440 µg/kg) had an ivermectin concentration above the MRL (10 µg/kg), and 68 samples below the LOD (10 µg/kg). For the liver, 1 sample (340 µg/kg) had an ivermectin concentration between the LOD (10 µg/kg) and the MRL (800 µg/kg) and 45 samples below the LOD (10 µg/kg). For meat, all samples had ivermectin concentrations under the MRL (30 µg/kg) and LOD (10 µg/kg).

Considering (1) the body weight (bw) of a person (man or woman) under 10 years of age (Avg. 13.49) and of a person (man or woman) over 10 years of age (Avg. 68.39), (2) the individual consumption data, (3) and the results of the estimated amount of ivermectin, the estimate daily intake (EDI) of ivermectin residues through milk, meat, and liver was close to zero (between 0.02 and 0.0935 µg/kg bw/day), i.e., the lower limit of the ADI (0 μg/kg bw), and also largely lower than the upper limit of the ADI (10 μg/kg bw) [15]. Furthermore, the GECDE for ivermectin residues was between 0.0029% (Scenario A) and 0.2959% (Scenario B) of the ADI for a person older than 10 years, i.e., lower than the 7.2% recommended. In addition, the GECDE for ivermectin residues was between 0.9131% (Scenario A) and 1.0246% (Scenario B) of the ADI for a person younger than or equal to 10 years, i.e., also largely lower than the recommended level of 9.3% [15] (Table 2). Complementary stochastic modelling confirmed the same picture, with only 1 EDI simulation among the 10,000 simulations with values higher than the ADI for a person younger than or equal to 10 years of age. Indeed, the expression of the risk should be qualified as rare to very rare. Additionally, despite the maximum residue limit (MRL) being set to 10 µg/kg for ivermectin, the results show common use of the drug, with reported residue levels in positive samples between 5.00 and 38.50 µg/kg, at least in some milk samples. While population-level exposure may remain under the MRL, the situation becomes alarming for children. Given their lower body weight (below 10 kg) and, for instance, the recommended daily milk consumption of 0.42 kg, their exposure greatly exceeds the MRL, raising concerns about milk safety, particularly from small-scale farms. This finding suggests a need for stricter regulations and enforcement in milk production to protect vulnerable populations like children [56].

Although no long-term toxicity studies have been conducted with repeated doses in humans or other laboratory animals, studies with abamectin in mice (94 weeks) have found carcinogenic effects [57]. In addition, short-term studies (4 weeks) in young rats have shown increased sensitivity to ivermectin due to an underdeveloped blood–brain barrier [58]. Given its recent use during the COVID-19 pandemic, clinical effects, including neurotoxicity, gastrointestinal symptoms, and musculoskeletal complaints, have been reported. Patients taking high doses of veterinary ivermectin reported neurotoxicity, with altered mental status. On the other hand, patients taking lower doses of ivermectin over a prolonged period reported milder toxicity, with no cases of severe altered mental status [59].

It is important to note that milk consumption in the study areas was about a quarter of the national average. This suggests that risk assessments could yield different results in regions with higher milk consumption. While no immediate risk was identified, the potential danger remains, and measures should be implemented to ensure food safety. Given that milk is a basic product in the basic food basket and provides essential micro- and macronutrients, particularly crucial during infancy and childhood, when bone mass growth is critical, ensuring its safety is paramount [60].

There may be other potentially more dangerous scenarios, such as the consumption of milk from cows recently treated with ivermectin, particularly if consumed during the short-term withdrawal period. While this study focused on the mean consumption of foods of bovine origin in the study areas, the scenario mentioned is certainly plausible among small farmers, where the accumulation of milk consumption over several days could lead to hazardous or toxic situations.

Given that, currently, only 56% of the 823 formal slaughterhouses in operation meet the Slaughterhouse Under Official Inspection (MABIO) certification standards, which guarantee that the protein is safe and processed in authorised facilities [61] and that around 36% of the meat consumed in households and restaurants comes from informal slaughterhouses [50], this study does not guarantee food safety for consumers of beef protein in the area studied. It therefore recommends that future studies employ a more comprehensive sampling strategy, including samples from local butchers and a larger number of samples. On the other hand, not all provinces in Ecuador have official livestock slaughterhouses, forcing small and medium livestock farmers to incur higher costs to transport their animals to neighbouring provinces, sell their livestock at lower prices to intermediaries, or resort to home or clandestine slaughter, making it even more difficult to have a traceability system in place. All this underlines the need for governments or industry stakeholders to be able to offer financial incentives to small-scale producers who demonstrate compliance with withdrawal periods and good agricultural practices. In addition, the creation and improvement of active slaughterhouses and the updating of inspection programmes to include residue testing for pesticides, antibiotics, and heavy metals will help ensure safe and high-quality food for Ecuadorian consumers. Equally important are training programmes for farmers and informal market vendors, which can raise awareness about the importance of withdrawal periods and food safety, thereby improving compliance across the sector.

## 4. Conclusions

This study provides critical insights into ivermectin residue levels across various bovine products and excretions, with the most significant findings observed in faeces. The mean ivermectin residue in the liver (16.46 µg/kg) and meat (5 µg/kg) remained within the acceptable limits set by the JECFA (LMR = 800 µg/kg and 30 µg/kg, respectively). However, in milk, residues (12.46 µg/kg) exceeded the permitted MRL (10 µg/kg), raising concerns about food safety compliance. Despite this, the risk from chronic dietary exposure was deemed low, with the likelihood of adverse health effects considered rare to very rare. While the health risks from consuming products such as meat, liver, and milk appear minimal under normal conditions, this study highlights the potential dangers of scenarios such as consuming milk from cows recently treated with ivermectin, especially during the short-term withdrawal period. This raises important questions about the regulation of antiparasitics in livestock and the safety of dairy products particularly from small-scale farms. This finding suggests a need for stricter regulations and enforcement in milk production to protect vulnerable populations like children. Additionally, given the high presence of ivermectin in faecal samples, from one health perspective, the presence of residues in faeces poses potential environmental hazards, warranting further research. This study emphasises the importance of collaborative and intersectoral efforts. Veterinary professionals, public health experts, biologists, and ecologists must work together to address this issue, ensuring good animal health, food safety, and human health through sustainable and environmentally friendly livestock practices.

## Figures and Tables

**Table 1:** Daily consumption of meat, liver, and milk in g/person/day.

Product	Households (%)	Less or Equal to 10 Years Old			Higher than 10 Years Old		
		Consumers (%)	DC ^A^	DC ^B^	Consumers (%)	DC ^A^	DC ^B^
Milk	97	98	94.76	98.24	97	95.85	102.15
Meat	91	91	5.88	6.45	92	30.95	35.62
Liver	30	32	0.14	0.35	30	0.75	2.27

Legend. (DC) Daily consumption (grammes): (A) scenario with the mean consumption of the inhabitants of the study area; and (B) scenario with the mean consumption only of the inhabitants that consume each food of bovine origin.

**Table 2:** Daily intake of ivermectin and chronic dietary exposure.

		Food of Bovine Origin	Median Residue Concentration (µg/kg)	Mean Residue Concentration (µg/kg)	Consumption Percentile 97.5th (kg/day)	Consumption Means (kg/day)	bw (kg)	EDI (µg/bw/day)	EDI (µg/kg bw/day)	Exposure (µg/kg bw/day)		GECDE	
										97.5th	Mean	µg/kg bw/day	%ADI
	Less or equal to 10 years old	Milk	5.0000	12.4571	0.2404	0.0948	13.4935	1.1804	0.0875	0.0891	0.0351	0.0891	0.8908
A		Meat	5.0000	5.0000	0.0118	0.0059	13.4935	0.0294	0.0022	0.0044	0.0022	0.0022	0.0218
		Liver	5.0000	16.1667	0.0008	0.0001	13.4935	0.0022	0.0002	0.0003	0.0001	0.0001	0.0005
		TOTAL						1.2120	0.0899	0.0937	0.0373	0.0913	0.9131
	Higher than 10 years old	Milk	5.0000	12.4571	0.3668	0.0958	68.3891	1.1940	0.0175	0.0268	0.0070	0.0268	0.0027
A		Meat	5.0000	5.0000	0.0724	0.0309	68.3891	0.1547	0.0023	0.0053	0.0023	0.0023	0.0002
		Liver	5.0000	16.1667	0.0056	0.0008	68.3891	0.0122	0.0002	0.0004	0.0001	0.0001	0.0000
		TOTAL						1.2609	0.0200	0.0325	0.0093	0.0291	0.0029
	Less or equal to 10 years old	Milk	5.0000	12.4571	0.2697	0.0982	13.4935	1.2238	0.0907	0.0999	0.0364	0.0999	0.9993
B		Meat	5.0000	5.0000	0.0133	0.0065	13.4935	0.0323	0.0024	0.0049	0.0024	0.0024	0.0239
		Liver	5.0000	16.1667	0.0011	0.0004	13.4935	0.0057	0.0004	0.0004	0.0001	0.0001	0.0013
		TOTAL						1.2618	0.0935	0.1053	0.0389	0.1025	1.0246
	Higher than 10 years old	Milk	5.0000	12.4571	0.3668	0.1022	68.3891	1.2725	0.0186	0.0268	0.0075	0.0268	0.2682
B		Meat	5.0000	5.0000	0.0733	0.0356	68.3891	0.1781	0.0026	0.0054	0.0026	0.0026	0.0260
		Liver	5.0000	16.1667	0.0072	0.0023	68.3891	0.0367	0.0005	0.0005	0.0002	0.0002	0.0017
		TOTAL						1.4873	0.0217	0.0327	0.0102	0.0296	0.2959

Legend. (A) Scenario with the mean consumption of the inhabitants of the study area; (B) scenario with the mean consumption only of the inhabitants that consume foods of bovine origin; estimated daily intake (EDI); acceptable daily intake (ADI); global estimated chronic dietary exposure (GECDE); and body weight (bw).

## Data Availability

The original contributions presented in the study are included in the article/Supplementary Materials, further inquiries can be directed to the corresponding authors.

## Supplementary Materials

The following supporting information can be downloaded at: https://www.mdpi.com/article/10.3390/foods13213470/s1, Supplementary File S1: HPLC Protocol. References [62,63] are cited in the Supplementary Materials.
