# Supplementary material for: Evaluating the Human Risks of Consumption of Foods of Bovine Origin with Ivermectin Residues in Ecuador
Source: Foods. 2024 Oct 29;13(21):3470. doi: 10.3390/foods13213470 (PMC11545660; doi:10.3390/foods13213470)
Supplement: Supplementary file 1 [file foods-13-03470-s001.zip › foods-3212057-supplementary.pdf]

DOCUMENT S1: DETERMINATION OF IVERMECTIN CONCENTRATION BY USING THE HPLC CHROMATOGRAPHIC METHOD

1. OBJECTIVE

Establish the necessary guidelines for the determination of the concentration of ivermectin (B1a) by HPLC chromatography.

2. SCOPE

It is applicable to products and matrices containing ivermectin such as veterinary products, soil, food and biological matrices.

3. PRINCIPLE

It consists of the extraction of ivermectin using the HPLC chromatographic method, for subsequent identification and quantification.

4. SAMPLE PREPARATION

- Sample handling is carried out according to the procedure Instructions for Transport, Handling, Reception, Protection, and Disposal of samples IG- 01-5.8.
- Homogenise the sample by shaking the container before opening and start the Ivermectindetermination.

5. LIMITS

- Limit of quantification: < 100 µg /kg
- Limit of detection: < 10 µg/kg

$$LOD = \frac{Conc.\left(\frac{\mu g}{mL}\right) x\ quantity\ of\ mobile\ pahse\ (mL)}{weight\ of\ sample\ (g)}\ x\ 1000$$

$$LOD = \frac{0.004\ \frac{ug}{mL} x \times 25mL}{10\ g}\ x\ 1000$$

6. WORK RANGE

Not applicable

7. EQUIPMENT

- Analytical Balance Boeco BXX 31
- HPLC Perkin Elmer Serie 200 with JASCO UV-975 detector
- ZORBAX Eclipse Plus C18.5 µm column, 4.5 x 150mm CI-058
- Ultrasonic Cleaner PS-40A
- 5mL, 10 mL, 25 mL and 50 mL volumetric flasks
- Filter paper

- HPLC vials
- Micro filter pore 0.45 μm

8. TECHNICAL SPECIFICATIONS OF EQUIPMENT

See equipment list Physical-Chemical Area L-01-5.5

9. REAGENTS

- Water grade I
- Methanol
- Acetonitrile

Preparation of Mobile Phase: Prepare a mixture of acetonitrile, methanol and water (53:27:20) v/v with the aid of a measuring cylinder.

10. REFERENCE MATERIALS

Ivermectin standard

11. ENVIRONMENTAL CONDITIONS

- Temperature: Upper limit 35°C
  Lower limit 15°C
- Humidity: Upper limit 70%.
  Lower limit 25%.

See Environment and Equipment Control Record R-01-5.3

12. USE AND VERIFICATION OF EQUIPMENT

- See Instructions for the use of the IA-FQ-02 scale.
- See HPLC IA-FQ-15 Instructions for Use

13. SAFETY REGULATIONS

- Check that the balance is calibrated, clean and in perfect condition.
- Check that the HPLC is in proper condition before proceeding with the test.

14. PROCEDURE

14.1 PREPARATION OF THE STANDARD

Weigh about 25 mg of Ivermectin standard in a 25 mL volumetric balloon and dissolve with the mobile phase. Ultrasonate for 10 minutes or until completely dissolved. Remove and cool it, then bring it to volume with the mobile phase.

Make serial dilutions from the stock solution by taking an aliquot of 1 mL in a 10 ml balloon and making a volume dilution with mobile phase (D1). Carry out the same procedure until a dilution of 1/10000 (D4) is reached. This solution corresponds to standard 4.

From D4, take aliquots of 2, 3, and 4 mL in 10 mL beakers and make up the volume with mobile

phase, the latter corresponding to standards 1, 2, and 3, respectively. From dilution 1/1000 (D3), take an aliquot of 5 mL in a 10 mL beaker and make up the volume with the same diluent as for standard 5.

Principle	Standard	Theoretical Concentration (µg/mL)
Ivermectin	1	0.02
	2	0.03
	3	0.04
	4	0.10
	5	0.50

Directly filter approximately 1 mL of the samples to 0.45 µm membrane HPLC vials, discarding the first 2 mL of the filtrate.

14.2 SAMPLE PREPARATION:

- Weigh approximately 1 to 2 g of sample into a 5 mL liquid sample cup.
- Add about 2 mL of mobile phase and ultrasound for 10 minutes. For solids, weigh 5 to 10 g of sample in a 25 mL balloon. Add mobile phase and ultrasonicate for 10 minutes.
- Remove from the ultrasound, allow to cool and bring to volume with mobile phase, filter and microfilter through a 0.45 µm pore microfilter and inject into the apparatus.

14.3 CHROMATOGRAPHIC CONDITIONS:

Column:	ZORBAX Eclipse Plus C18 5µm, 4.5 ^150mm CI-058
Oven temperature:	30°C
Mobile Phase:	Acetonitrile: Methanol: Water (53:27:20)
Flow rate:	1.5 ml/min
UV detector:	245 nm
Injection Volume:	190 µm

15. REPORT OF RESULTS

The results of the analysis are reported as ppb in the Physical-Chemical Area Results Record R-03-4.1 of the corresponding Work Order.

The correlation coefficient, the slope, and the intercept are obtained from the curve (see Annex A—Calibration). The latter two were used to calculate the Ivermectin content in the samples, taking into account the dilutions to which the sample was subjected and the weighed portion.

Using the following expression, the corresponding concentrations or each sample expressed as ppb (µg/Kg) are determined:

$$ppb = \frac{(AUC_{sample} - Intercept) * volume\ 1 * 1000}{Slope * \ mass_{sample}}$$

Where:

- AUC $_{Sample}$: Area under the curve corresponding to the sample.
- Capacity: Capacity of the sample in mL
- Mass $_{Sample}$: Weight of each sample taken, expressed in g.

16. QUALITY CONTROL

Not applicable

17. ACCEPTANCE AND REJECTION CRITERIA

Not applicable

18. UNCERTAINTY OF THE METHOD

Not applicable

19. REFERENCES

USP 42, Official Monographs, Ivermectin, Pp. 2555-2556.

Nuñez M., Palma M., Araneda M., & Pérez R., (2007), Validation of an analytical method and determination of ivermectin residues in sheep tissues. Rev. Cient (Maracaibo) v. 17 n.6.
